# Fabrication, Polarization of Electrospun Polyvinylidene Fluoride Electret Fibers and Effect on Capturing Nanoscale Solid Aerosols †

**DOI:** 10.3390/ma9080671

**Published:** 2016-08-09

**Authors:** Dinesh Lolla, Manideep Lolla, Ahmed Abutaleb, Hyeon U. Shin, Darrell H. Reneker, George G. Chase

**Affiliations:** 1 Department of Chemical and Biomolecular Engineering, The University of Akron, Akron, OH 44325, USA; dl62@zips.uakron.edu or dineshlolla@gmail.com; 2 Larsen & Toubro India, Hyderabad 500018, India; manideep.lolla1711@gmail.com; 3 Department of Chemical Engineering, Jazan University, Jazan 45142, Saudi Arabia; azabutaleb@jazanu.edu.sa; 4 Hyundai Motor Company, Seoul 18280, Korea; hushin@hyundai.com; 5 Department of Polymer Science, The University of Akron, Akron, OH 44325, USA; reneker@uakron.edu

**Keywords:** electret, electrospinning, polarization, polyvinylidene fluoride, filtration

## Abstract

Electrospun polyvinylidene fluoride (PVDF) fiber mats with average fiber diameters (≈200 nm, ≈2000 nm) were fabricated by controlled electrospinning conditions. These fiber mats were polarized using a custom-made device to enhance the formation of the electret β-phase ferroelectric property of the fibers by simultaneous uniaxial stretching of the fiber mat and heating the mat to the Curie temperature of the PVDF polymer in a strong electric field of 2.5 kV/cm. Scanning electron microscopy, Fourier transform infrared spectroscopy, thermal gravimetric analysis, differential scanning calorimetry and Brunauer-Emmett-Teller (BET) surface area analyses were performed to characterize both the internal and external morphologies of the fiber mat samples to study polarization-associated changes. MATLAB simulations revealed the changes in the paths of the electric fields and the magnetic flux inside the polarization field with inclusion of the ferroelectric fiber mats. Both polarized and unpolarized fiber mats were challenged as filters against NaCl particles with average particle diameters of about 150 nm using a TSI 8130 to study capture efficiencies and relative pressure drops. Twelve filter experiments were conducted on each sample at one month time intervals between experiments to evaluate the reduction of the polarization enhancement over time. The results showed negligible polarization loss for the 200-nm fiber sample. The polarized mats had the highest filter efficiencies and lowest pressure drops.

## 1. Introduction

Air pollution over the planet is presently one of the most significant human health concerns. The World Health Organization (WHO) estimated in 2014 that about seven million premature deaths were a direct result of the exposure to pollutants in air (more than one out of eight global deaths) [1]. Rapid industrialization, rocket exhaust and dust storms are potential sources of human health hazards. New medical research has linked exposure to significant amounts of pollutants, including particulates, in air to cardiovascular diseases, heart strokes and many types of cancers [2,3,4,5]. Research and development of air filtration systems has gained immense importance in the past decade with the rise of global pollution, for indoor air filtration devices and industrial protective equipment. Traditional air filtration media have relied on a variety of materials, ranging from cellulose to advanced glass microfibers [6,7,8,9,10]. Air filters are characterized by their ability to remove particles from air streams (capture efficiency) and the associated pressure drop (resistance to air flow).

The use of sub-micron-sized polymer fibers in filter media is expanding due to improved capture efficiencies compared to larger diameter fibers [10,11]. Electrospinning is capable of producing continuous long single fibers with relatively small diameters ranging from a few nanometers to about 10 microns. Sub-micron fibers have high surface areas per unit mass and small pore sizes that correlate with the greater capture efficiencies of small particles, but at the expense of a higher pressure drop. Electrospinning parameters can be varied to alter the properties of the fibers and fiber mats (fiber diameters, internal porosity, surface charges and formation of beads), which may affect filter performance.

Electrospinning is one of the most frequently-reported techniques in the past two decades to derive slender three-dimensional, multilayered structured non-woven fiber mats from many synthetic polymers. It gained research and commercial interest due to its simplicity, ease of maintenance and low cost of production of small quantities [12,13]. Electrospinning uses a high voltage DC current to generate electrostatic repulsions inside of droplets of polymers mixed with a volatile solvent. When the electrical repulsion forces exceed the surface tension forces, a thin continuous polymer jet launches from the drop. The polymer jet elongates and tapers as it travels towards a grounded collector in a circular-pendulum motion. These motions were caused by bending instabilities due to the viscoelastic properties of the polymer solution [14,15].

Electrospinning is governed by many process and material parameters, which significantly affect the fiber size and morphology. Thompson, et al. [16] identified thirteen potential parameters, which affect fiber diameters, and evaluated a theoretical model to interpret their individual effects. The change in ambient conditions, such as humidity and temperature, also affects the fiber external morphology and bead formation phenomena. Low production rates limited by the rate at which polymer solution is carried by a single jet are a limitation for the scale-up of the process for bulk-scale industrial manufacturing. The most economical way of addressing the production rate is by using multiple nozzles. Needleless electrospinning techniques have also been developed, but consume higher Direct Current (DC) and produce fibers with relatively larger diameters [17]. 

Polyvinylidene fluoride (PVDF) is a semi-crystalline polymer with a monomer unit of (CH_2_-CF_2_) that has a planar zig-zag carbon chain with alternating two fluorine atoms attached to one carbon and two hydrogen atoms attached to the adjacent carbon. Since fluorine atoms are electronegative and the hydrogen atoms easily share electrons, this creates strong external dipole forces that are perpendicular to the axis of the individual molecules. PVDF is well known for its piezo-, pyro- and ferro-electrical properties [18,19,20]. In addition to its electrical properties, it is available in five crystalline phases α, β, γ, δ and ε in which the polar β-phase has attracted the most research attention due to relatively high spontaneous polarization and net dipole moment [21,22]. When electro-mechanically stretched, the polymer molecules in PVDF align themselves in α and β conformations [23]. PVDF is lightweight, flexible and has the potential to hold a static charge for long durations due to its peculiar molecular structure. IR absorption vibrational spectra provide information on the internal conformational geometry [24,25,26].

Slender electrospun polymeric fibers, due to their high surface to volume ratios and well defined internal pore structures, have become one of the most advanced materials in the air filtration industry. Filter media with charged fibers are termed “electrets” [27,28]. In general, an electret is defined as a permanently polarized and electrically insulating material with long-lived internal and/or external quasi-permanent surface charge [29]. For non-electret media, the dominant mechanisms for the capture of nano- and micro-scale particles from air streams are interception and Brownian diffusion. Electret or electrostatic fiber media also possess an electrostatic attraction mechanism, which allows the fabrication of media with larger pore openings, hence lower pressure drop, but maintains high capture efficiency [30,31]. The projection of the electrical fields from the fibers into the pore openings is necessary for the electrostatic attraction mechanism to be effective. The stronger the electric field, the farther the effect extends into the pore spaces and the more effective is the mechanism for particle capture. For PVDF fibers, the dipole moments are distributed along the fiber axis, and the electrostatic attractive forces integrate along the length of the fiber segments [32]. This integration of the electrostatic forces strengthens the electric field projected from the fibers.

Electrets can be made from a number of polymers, including fluoropolymers, poly propylene and poly(ethylene terephthalate) [33]. Malti [34] reported the description of electret formation in dielectric materials and studied polarization-induced changes by piezoelectric-induced pressure pulse (PIPP) to reconstruct the three-dimensional charge distribution in electrets and applications as air filters and transducers. Ferroelectric fibers of polymeric materials were used as an alternative for high efficiency particulate air filters (HEPA) in nuclear fuel separations of particles with a size of 1–10 μm [35]. Emi, et al. [36] experimentally investigated the transient behavior of aerosol filtration in several layers of 200 mesh wire screens in fibrous model filters, and they observed an increase in pressure drop with the increase in collection efficiency with time.

Milena, et al. [27] studied the charge storage capacity of poly(ethylene terephthalate) for a short span of 50 days in fibers produced by electrospinning and the corona discharge method. They determined that fibers produced by the corona discharge method had a higher value of the storage charge over electrospun fibers. Kilic, et al. [37] enhanced the electret properties of melt-spun poly propylene composite filaments of diameters of about 20 μm using nonlinear dielectric materials like barium titanate. They performed charge decay tests on cold charged and thermally-charged pp filaments at 130 °C and measured surface potentials after 0.5 h, 1 h, 2 h, 4 h, 8 h, 12 h, 16 h and 24 h decay times. To our knowledge, electret PVDF micron and submicron fiber mats have not been tested for long time charge decay analysis for use as filter media with particle accumulation.

The advantages of electret fiber media are higher capture efficiency and lower pressure drop than non-electret fiber media of similar fiber sizes and mat structure. The disadvantages of electret media are the shelf life of the electrostatic charges and during filtration, the neutralization of static charge by the capture of oppositely-charged particles. When the charge is reduced or neutralized, then the dominant capture mechanisms become direct interception and Brownian motion, and the electret media perform the same as a non-electret material of similar fiber sizes and fiber structure. Electrostatic charge build up in electret media may cause sparks that can cause dust explosions or ignite [38].

Salimi, et al. [39] performed polarization by uniaxial stretch heating without an external electric field on compression-molded PVDF films of different grades with about 35% crystallinity. Their investigations using DSC revealed a notable increase up to 43% crystallinity with 74% β-phase in polarized PVDF sheets. AFM characterizations on PVDF films revealed changes in surface roughness with applied electric field in real time; different peak-to-peak distances were observed with a nearly linear increase with external electric stimuli from 5–30 V [33]. Recent studies on ferroelectric and piezoresponse observed using piezoresponse force microscopy (PFM) showed individual electrospun PVDF fibers with diameters of about 70, 170 and 400 nm having 86.6%, 84.2% and 81.0% crystallinity due to higher conformational changes [23].

Filter media of electrospun fibers are known to perform better than microfiber media in terms of capture efficiency and pressure drop. A recent comparison of electrospun cellulose acetate fibers and commercial glass fiber media challenged with solid (NaCl) and liquid (diethyl hexyl sebacate oil) aerosols at high face velocities (45 cm/s) revealed that electrospun fiber media had higher filter indices [35]. Filter media with electrostatic charges on fiber surfaces can enhance particle capture and reduce the most particle penetrating particle size (MPPS) in the range of 150 nm, as compared to glass filter media of similar fiber sizes, but without electrostatic field enhancement [40,41,42,43].

In this work, PVDF fiber media were electrospun and conditioned to enhance the electrostatic properties. These media were tested to determine their performance to capture NaCl nanoparticles. These electret filters were repeatedly tested over a period of 330 days to observe the effects of charge loss over time. The results showed that the filter media retained their static charges and associated capture properties over the 11-month time, even when retaining particles from prior tests.

## 2. Experimental Description

### 2.1. Materials

PVDF, trade name Kynar with an average molecular weight of about 250,000, density 1.78 gm/cm^3^ and melting point between 165 and 172 (as per the MSDS), was supplied by Archema Inc. (King of Prussia, PA, USA). Acetone, *N*,*N*-dimethylformamide (DMF) and trifluoroacetic acid (TFA) were purchased from Sigma Aldrich (St. Louis, MO, USA) and were used without any further purification.

### 2.2. Electrospinning

The PVDF was dissolved in solvent blends of wt. %:wt. % ratios of DMF to acetone of 50:50 and 80:20. PVDF readily dissolved in acetone, but the high volatility of acetone by itself caused the fiber jet to solidify too rapidly during electrospinning and resulted in the formation of microfibers. The DMF solvent was used to reduce the solvent volatility to allow electrospinning of submicron fibers. The PVDF-acetone mixture was immiscible in DMF at room temperature, but was miscible at 70 °C. The polymer solutions were prepared in the indicated proportions and mildly stirred for 20 min. PVDF was added to the 50:50 solvent blend to form a 10 wt. % polymer solution and was added to the 80:20 solvent blend to from a 20 wt. % polymer solution, respectively.

The polymer solutions were stirred at 70 °C for 20 min until clear, transparent and homogenous solutions were formed. Trifluoroacetic acid (TFA) was added to the 50:50 solution at 3 wt. % of the mixture to increase the electrical conductivity of the solution to enhance the spinnability and reduce the formation of beads on fibers. The addition of NaCl, LiCl or MgCl_2_ ionic salts to a polymer solution can also serve the purpose of increasing conductivity, but they also decrease the surface tension [44]. For the polymer solutions in this work, any decrease in surface tension led to electrospraying rather than electrospinning [45].

**Figure 1:**
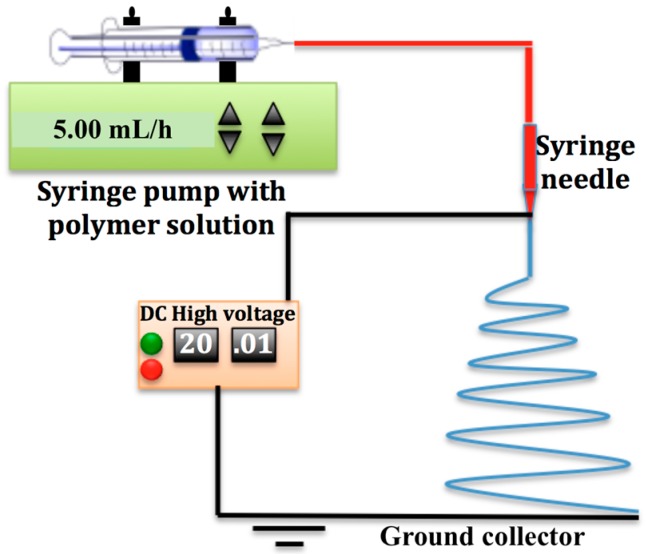
Schematic of the syringe pump electrospinning station with a rotating cylindrical collector.

In this work, the parameters that affect the fiber morphology were controlled to attain the desired fiber size distributions and to eliminate the formation of beads. Figure 1 is a schematic of the electrospinning setup used in this work. Five-milliliter plastic syringes were preheated to 70 °C, charged with polymer solution at 70 °C and connected by Teflon^TM^ tubing to a stainless steel 21-gauge blunt needle. The polymer solutions were pumped to the tip of the needle by a syringe pump (SP220i World Precision Instruments, Sarsota, FL, USA) with preset flowrates listed in Table 1. A high voltage DC power supply (ES60P, Gamma High Voltage Research) was used to generate the voltage potential listed in Table 1 between the syringe needle and the grounded non-stick aluminum foil on the rotating drum. The needle was incrementally moved by 2 cm to different positions along the axis of the rotating collector, with 5 mL of solution deposited at each position, until the drum surface was covered with a mat of fibers with an average specific weight of 20 g/m^2^. All of the electrospun sheets were heated for 2 h at 70 °C in an oven to evaporate residual solvents.

#### 2.2.1. Polarization of Electrospun PVDF Fibers

**Figure 2:**
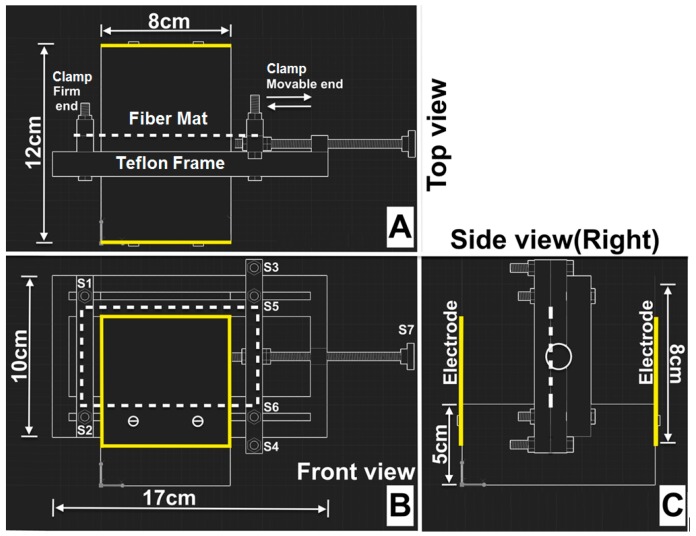
Perspective views of the custom-made polarization device designed using Autodesk^®^-AutoCAD^®^-2015. The orientation of the electrospun fiber mat is indicated by the dotted white lines, and aluminum electrodes are indicated by solid yellow lines. (**A**) Top view; (**B**) Front view; (**C**) Side view (right).

Polarization experiments for electrospun fiber sheets were performed using a custom-made polarization device made of a Teflon^®^ frame and 8 cm × 8 cm aluminum electrodes as marked in Figure 2. The Teflon was chosen over other materials due to its ease of machining and low electrical conductivity at the temperature range of the experiments. Electrospun fiber mats (indicated by the dashed lines in the figures) were cut into rectangles with dimensions of 10 cm × 6 cm for polarization. The 6-cm edges of the rectangular sheets were clamped at the firm and movable ends of the Teflon frame using screws (S1, S2, S3 and S4), shown in Figure 2B. The screw S7 was rotated to move the mobile clamp between screws S3 and S4 to stretch the fiber mats from the unstretched length of 10 cm to a stretched length of 11 cm. Screws S5 and S6 were tightened to fix the mobile clamp in the desired stretched position. The entire device was placed in a furnace (Model 13, Baker Furnace Inc., Yorba Linda, CA, USA) and heated at a constant ramp rate of 20 °C/min up to the Curie temperature of 150 °C then held at the Curie temperature for 20 min. The furnace was cooled down to room temperature at 20 °C/min.

During the heating and cooling, the two electrodes were electrically charged to an electric potential difference of 30 kV DC between the electrodes. The electric field generated in this geometry, approximately 30 kV/12 cm (2.6 kV/cm), was perpendicular to the surface of the fiber mat. The electric potential was applied via the same DC power supply used for the electrospinning by passing the electrical wires through the flexible insulation around the furnace door.

#### 2.2.2. Modelling of the Polarization Device Showed in Section 2.3.1

**Figure 3:**
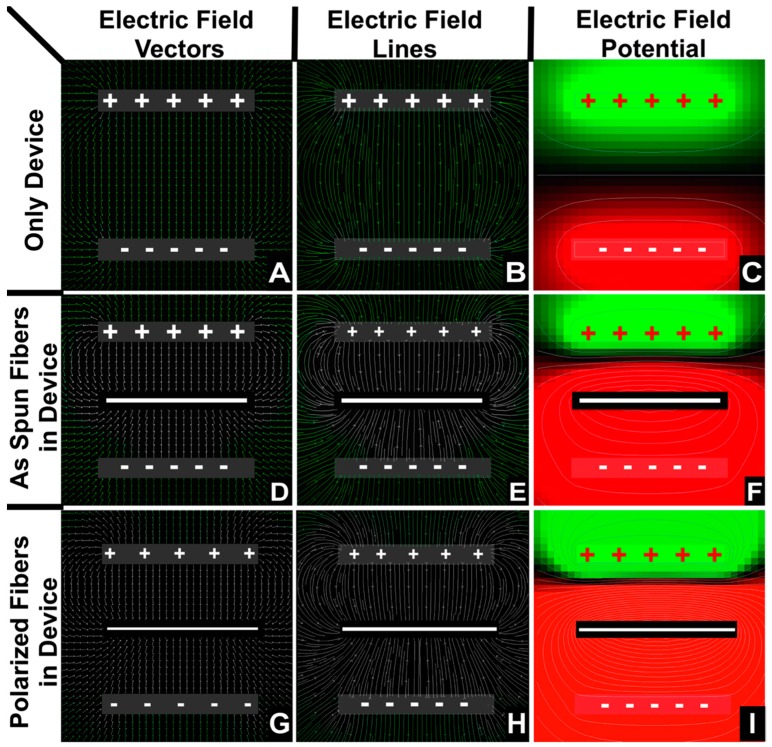
2D simulations of electric field current density vectors (**A**,**D**,**G**), field lines (**B**,**E**,**H**) and the contour plots of electric potential (**C**,**F**,**I**). The plots in (**A**–**C**) are the simulation results without the PVDF fiber mat. The plots in (**D**–**F**) are simulation results with the as-spun unpolarized PVDF fiber mat present. Plots in (**G**–**I**) are the results with the polarized PVDF mats present. The calculations were extended for 30 min with the polarization conditions mentioned earlier. The plus and minus symbols indicate the aluminum electrodes, and the solid white line at the center indicates the PVDF fiber mat orientation.

The polarization device showed in Figure 2 was modeled and the results plotted in Figure 3. To help interpret the polarization effects, the polarization device was modeled with and without fiber mats. The electric potential field was modelled by solving Gauss’s law for current density vector, *j*, similar to prior model calculations of electric fields around electrospinning jets [16,46]. Calculations were performed with respect to scale, but the thickness of the electrodes and fiber mat images in Figure 3 were enlarged for easier visualization. These calculations and plots were generated using MATLAB^®^ (The MathWorks Inc., Natick, MA, USA). Temperature, time, electric field density, conductivity and dielectric coefficient of PVDF fibers and air properties were taken into consideration for calculating electric field and potentials in Figure 3.

An electric field vector defines the direction and magnitude of the electric field at a given point. Electric field lines are lines drawn through the tangents of electric field vectors. They have a direction, but no magnitude in and of themselves. Field line magnitude is indicated by the inverse of distance between the field lines.

In a parallel plate capacitor, the electric field of lines travel in the direction from the positively-charged electrode to the negatively-charged electrode, as shown in Figure 3A–C. The fibers of a ferroelectric polymer material contain dipoles (two equal and opposite charges separated by a small distance) inside the fibers and are aligned in random directions. When a ferroelectric fiber mat is placed between the two electrodes with the application of a strong electric field and at the Curie temperature, the majority of the dipoles align in a particular direction, resulting in net polarization. The surface of the fiber mat nearest to the positively-charged electrode becomes negatively-charged and vice versa. Hence, there is a net change in the electric field lines due to the ferroelectric fiber mat as deduced by the comparison of Figure 3B,E. The amount of change in the electric field depends on the strength and concentration of the dipoles in the ferroelectric material. The total current between the electrodes gives a measure of the strength of the polarization of the fiber mats.

### 2.3. Characterization

#### 2.3.1. Morphology Analysis

The electrospun fiber morphologies were characterized using field emission scanning electron microscopy (EF-SEM, JSM-7401F JEOL Ltd., Peabody, MA, USA). A low accelerating voltage of 2 kV and stable high vacuum of 9.63 × 10^−5^ Pa was used to operate the SEM. All of the fiber samples were silver coated for 90 s using the EMITECH K575x Turbo Sputter Coater (Kent, UK). The thicknesses of the silver coatings on the fibers were less than 5 nm. Since polymer fibers have low conductivity, the sputter coating is commonly used to increase the conductivities of the specimens to improve the electron propagation through the sample. The coating also acts as a protective barrier on the fiber surfaces to minimize electron beam damage. Care was taken to mitigate the sample-charging phenomena by reducing the radiation exposure time, as the fibers with smaller diameters were more sensitive to damage by electron radiation. Fiber size distributions were calculated using FibraQuant^®^ 1.3 software (nanoScaffold Technologies LLC, Chapel Hill, NC, USA), using over 15,000 data points collected from multiple SEM images from 3 different samples.

Brunauer-Emmett-Teller (BET) surface area analysis was used to measure the surface areas and surface porosities of 0.5-g samples of fiber mats by liquid nitrogen gas absorption using a surface area and porosity analysis instrument (Micromeritics Tristar II, Micrometrics, Norcross, GA, USA). Fiber samples were filled into the spherical bottom of cylindrical glass tubes with filler rods, vacuum-sealed using a rubber stopper and dried for 5 h in a degassing station at room temperature before the analysis.

Molecular bonding and phase information of the samples were determined using a Fourier transform infrared spectrometer (Thermo Fisher Scientific Model: Nicolet iS50 FT-IR, Waltham, MA, USA). A small mat of fibers 1 cm × 1 cm was positioned between two KBr disks with care to not wrinkle the samples. IR spectra were recorded over the range of 400–5000 cm^−1^ at 8 cm^−1^ resolution, and 64 step scans were obtained for signal averaging.

#### 2.3.2. Thermal Analysis

Thermogravimetric analysis (TGA) was performed using TA Instrument Model TA Q50 (TA Instruments-Waters L.L.C. New Castle, DE, USA) under nitrogen gas environment. A 10-g sample was tested to determine the percentage of mass lost as a function of time and temperature. All of the samples were heated from room temperature to 800 °C at a 10 °C/min ramping rate.

Differential scanning calorimetry (DSC) was performed to analyze melting points, crystallization temperature and the percentage crystallinity, using TA Instrument Model Q200 (TA Instruments-Waters L.L.C. New Castle, DE, USA) under liquid nitrogen environment. One-gram samples were tightly sealed in stainless steel pans (one empty reference pan and one sample pan were used for each sample), and all of the samples were heated from −50 °C–200 °C at a ramping of 10 °C/min. Two cycles of heating and adiabatic cooling were performed; however, data points from the first cycle were discarded for precision.

#### 2.3.3. Electrostatic Analysis

Static surface charges on the fiber sheet were measured using a compact handheld electrostatic field meter (Simco-Model FMX-003, Simco ION, Hatfield, PA, USA). This device was capable of measuring static voltages within a range of ±20 kV with an accuracy of ±3%. The probe was held 2.54 cm away from the fiber mat using two LED guide rings for accuracy and ten readings from each sample were collected from different surface locations. Initial readings were set to zero to tare the static charges in ambient air. All samples were stored individually in aluminum foil for time-lapse calculations.

#### 2.3.4. Filtration Performance Analysis

Aerosol filtration experiments were performed using a TSI Model 8130 automated filter tester (TSI Incorporated, Shoreview, MN, USA) [47,48]. A 3 wt. % sodium chloride solution was used for generating the solid particles in the size range of about 10–250 nm. Filter tests were conducted at 10–50 L/min flow rates on fiber samples 5.7 cm in diameter with 10-L/min increments in flow rate to observe fiber mat performances as filter media. Three individual samples of each filter type were tested for each flow rate with a rise time of 59 s for each experiment. Resistance (pressure drop) and the efficiency of the separation (efficiency = particles captured/particles challenged = 1 − outlet stream concentration of particles/inlet stream concentration of particles) were determined for each sample. All samples were tested ten times on fiber mat samples from different regions of the nanofiber sheet to account for spatial variations that may have occurred in sheet thickness or fiber properties during the preparation of the fiber sheets.

### 2.4. Solution Properties

**Figure 4:**
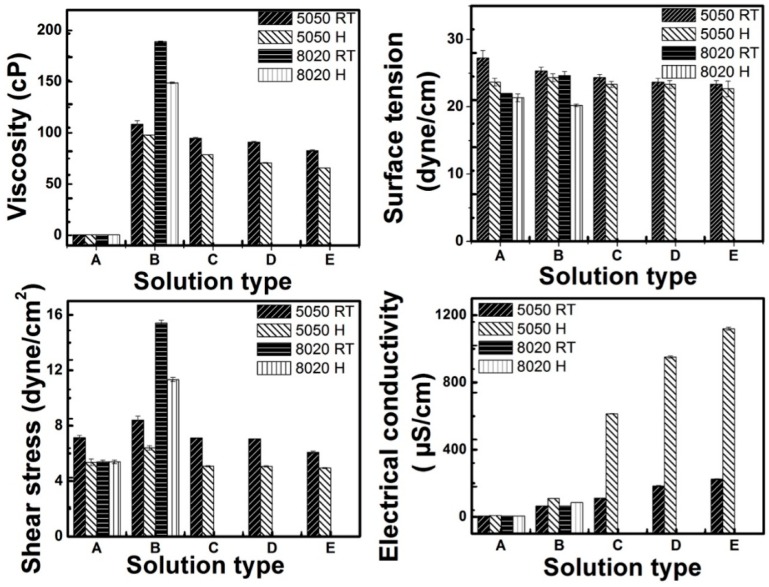
Properties of electrospinning solutions used to make fibers; the nomenclature on *Y*-axis represents: A, only solvent mixture; B, polymer and solvent mixture; C, D and E are 1%, 2% and 3% concentration of trifluoroacetic acid blended in to PVDF 5050 solution (10 wt. % of PVDF in 50 wt. %:50 wt. % of acetone:DMF); TFA was not added to the PVDF 8020 solution. RT, room temperature; H, heated to 70 °C.

The physical properties of the solutions reported in Figure 4 were measured at room temperature (RT) and at 70 °C (H). Ten readings were measured of each sample and averaged to reduce experimental error and one standard deviation indicated by the error bars. Electrical conductivities of the solutions were measured using an electrical conductivity meter (Tabletop Orion 3 Star, Thermo scientific, Waltham, MA, USA). The surface tension was measured with a CSC DuNouy Tensiometer (KrŰss, Hamburg, Germany) using a platinum ring.

The viscosity and shear stress of solutions were measured on 0.5-mL solution samples by a DV2T Model Brookfield viscometer with spindle 4 and 100 rpm speed. Sample temperatures during the measurements were controlled using a water bath heated at 25 °C and at 70 °C.

The histograms in Figure 4 show that the polymer solution properties were significantly different between 25 and 70 °C. At the higher temperature, the viscosities decreased, and the miscibility of the solutions increased to improve the blending of the mixture components. The changes in the electrical conductivities and viscosities of the solutions with the increase in temperature, with the addition of TFA, accounted for the improvement in the spinnability and fiber formation in the electrospinning at the elevated temperature.

## 3. Discussion of Results

### 3.1. Electrospun Fiber Characterization

**Figure 5:**
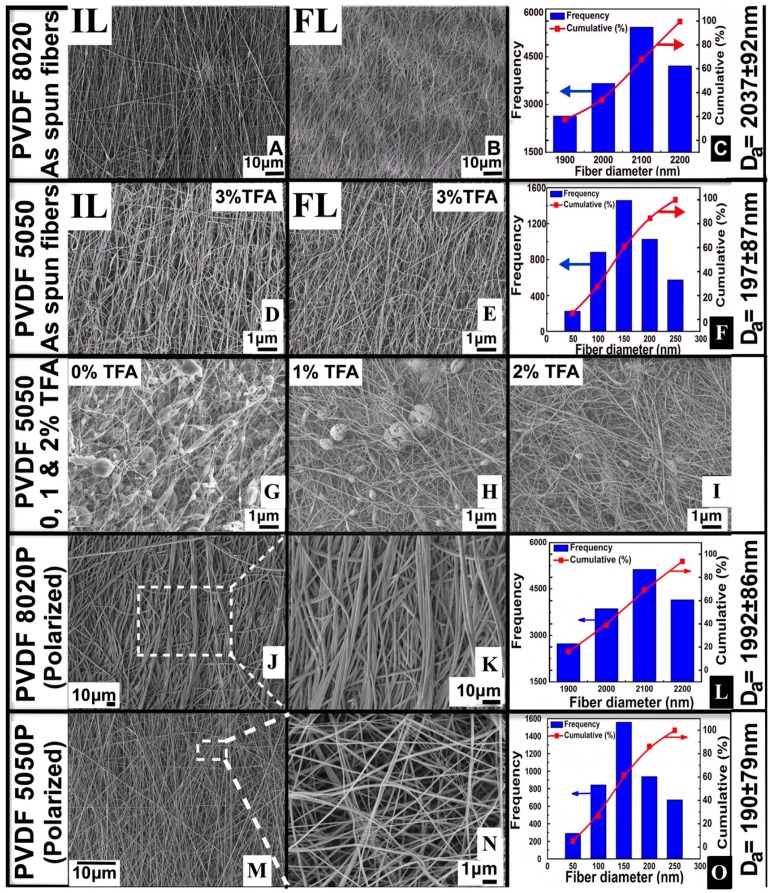
SEM images of electrospun PVDF fibers and the respective length-weighted frequency and cumulative distributions of the fiber size. The left-most column of each row shows “P” after the 5050 and 8020 notation for the solution composition percentages to indicate the polarized mats (**J**,**K**,**M**,**N**). The notations without the “P” indicate the as-spun non-polarized mats (**A**,**B**,**D**,**E**). The histograms in (**C**,**F**,**L**,**O**) represent fiber size distributions collected from nine SEM images. The average fiber diameters are given by D_a_. IL, initial layer (collector side); FL, final layer (front side). SEM images in (**G**,**H**,**I**) shows bead reduction analysis in PVDF 5050 fibers with addition of TFA.

SEM images of fiber mats are shown in Figure 5. The images in Figure 5A,B,D,E show the initial layers of fibers (side of mats in contact with the collector surface) and the final layers of fibers (side of mats facing the electrospinning jets during electrospinning). By inspection of the images, the fiber diameters appear to be similar on each side of the mats. Size distributions were calculated, shown in the plots in Figure 5C,F,L,O from the images of the final layer sides of the mats.

Comparison of the images in Figure 5A,D to Figure 5J,L show that the polarized and non-polarized fibers were about the same size. Fiber size distributions in Figure 5C,F are in agreement with Figure 5L,N. The fiber size distributions showed small decreases of the calculated average fiber diameter of the polarized fibers compared to the non-polarized fibers, possibly due to the evaporation of residual volatile solvent or polymer degradation. The average fiber sizes reported in Table 1 are averages of data points from 10 SEM images. For the non-polarized samples, front and backside images were measured.

Figure 5J,L shows low magnification SEM images of polarized PVDF fibers. Figure 5K,M is the high magnification SEM images of the rectangular areas highlighted with dotted lines in Figure 5J,L. LEI mode (lower secondary electron imaging mode) was used to image the surface morphology of fibers at high magnifications with a high depth of focus, which gave enhanced surface textural information. Polarized fibers showed conglutination phenomena as an effect of simultaneous mechanical, thermal and electrical treatment. Conglutination occurs where fibers are joined together at a point of contact. The contact between conglutinated fibers can be parallel or perpendicular depending on the alignment of the neighboring fibers. Surprisingly, there were many fibers with diameters significantly less than 50 nm distributed within the sample shown in Figure 5M. Such fibers commonly form as a result of thin secondary jets (see the Supplementary Material, Figure S2). 

Figure 5G–I shows images of the beads-on-fibers morphologies. As the TFA concentration increased, fewer beads were formed. Solutions with a low polymer concentration and higher DMF concentrations favored the formation of beads with diameters of about 1–5 μm in the electrospun fibers with the PVDF5050 solvent mixture without acid. A solution with 3% concentration of TFA in 10% of PVDF dissolved in 50 wt. %:50 wt. % of acetone:DMF at the electrospinning conditions given in Table 1 resulted in bead-free thin fibers, as shown in Figure 5D,E.

**Figure 6:**
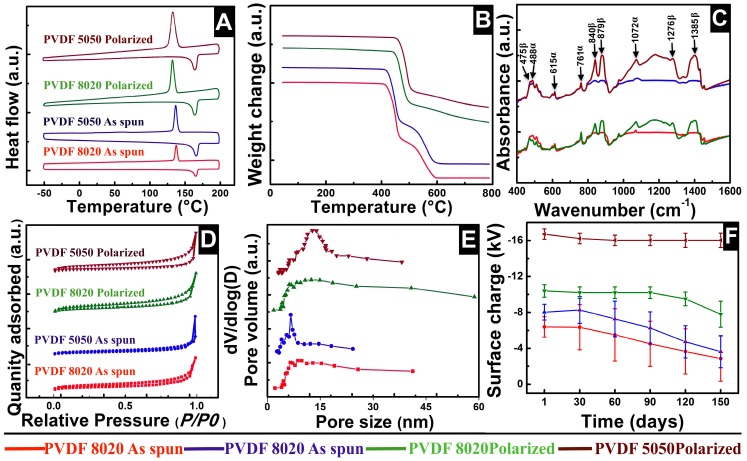
Characteristics’ comparison of PVDF fibers before and after polarization. The color correspondence indicated in (**A**,**D**) is the same in all of the plots. DSC (**A**); TGA (**B**); FTIR (**C**); BET (**D**); Surface porosity (**E**); Surface charge of PVDF fibers (**F**).

DSC (EXO-UP) profiles revealed that as-spun PVDF 8020 and 5050 fibers showed a comparatively lower percentage of overall crystallinity when compared to polarized samples, as shown in Figure 6A and Table 2. With respect to the degree of crystallinity of the samples, a significant change in the crystallization peak was observed. With the increase in crystallinity, the crystallization peak of the polarized fibers experienced a slight shift of about 5 °C towards the left, which is an indication of a lower amorphous state compared to unpolarized fibers.

TGA of all four samples with equal weights showed three-step weight loss patterns. Samples stayed unchanged without any weight loss up to 400 °C. Figure 6B shows that weight loss caused a pyrolysis reaction resulting in the first weight loss peak, attributed to CH_2_, and the second peak denotes CF_2_ degradation. Polarized samples possessed about 15% of leftover mass after heating to 800 °C indicating that they required higher amounts of thermal energy to degrade higher crystalline structures with more oriented CF_2_ repeat units, whereas unpolarized fibers completely degraded at ≈600 °C. The degradation temperatures of CH_2_ and CF_2_ weight losses are reported in Table 2.

Figure 6C is a comparison overlay of the IR spectra profiles in the fingerprint region between 400 and 1600 cm^−1^; data points from other bandwidths and KBr spectra were discarded for the comparison. KBr (alkali halide) was used as a substrate, since it does not show any absorption spectrum in the fingerprint IR region. All of the samples showed a mixed composition of both the α- and β-phase at nine distinct peaks. Peaks corresponding to bandwidths at 488, 615, 761 and 1072 cm^−1^ are associated with the α-phase, whereas peaks at 475, 840, 879, 1276 and 1385 cm^−1^ represent the β-phase. Peaks at low IR regions, such as 615 and 761 cm^−1^, belong to CF_2_ skeletal bending, which is a very common phenomenon in electrospun samples due to rapid mechanical stretching, and peaks at higher bandwidth regions 1020–1330 cm^−1^ are the result of macroscopic molecular moments due to spontaneous electrical polarization; these peaks were not so intense in unpolarized samples. Comparisons show the enhancement of the polarized β-phase, which is clearly evident from the highly intense peaks at 1276 and 1385 cm^−1^ on polarized fibers, and the individual composition of the β-phase in each sample was calculated using Gregorio’s correlation or Beer-Lamberts law using IR absorption bandwidths in ATR mode.

Figure 6D shows Brunauer-Emmett-Teller (BET) isotherm trends of liquid nitrogen adsorption and desorption. Nitrogen gas is considered the most suitable adsorbing agent for surface textural characterization of porous solids, especially multilayered samples. As-spun PVDF fibers showed similar profiles and followed Type 2 classical isotherm patterns for both of the samples; whereas polarized fibers also followed a Type 2 isotherm with a hysteresis loop during nitrogen desorption. Capillary condensation was responsible for the formation of the sorption hysteresis loop on the polarized fibers due to the enhanced surface pore sizes caused by thermally- and electrically-driven motions. Capillary condensation on the mesoporous structures, seen as the rate of desorption, was comparatively lower than the rate of nitrogen absorption due to multilayer adsorption on pore walls.

Figure 6E summarizes the surface pore size distributions within the fibers corresponding to isotherms in Figure 6D. Figure 6E is plotted against BJH desorption values; adsorbed nitrogen molecules desorb at different partial pressures depending on the pore size; the lower the pore size, the higher (P/P_0_) value. The low slope region at the center of the isotherm during both adsorption and desorption indicates that the sample was multilayered as the fibers were deposited one over the other. A slight decrease in overall surface areas of the polarized fibers were observed over the untreated fibers due to the fiber conglutination (see Table 2).

Figure 6F shows the average static charge over the surface of the fiber sheets. The strong CF_2_ dihedral bond on one side of the backbone of the polymer was likely the reason for the negative charge on the fibers, and this remained the same even when the polarity of the electrospinning needle was changed from +20 kV to −20 kV (all else remained the same). The polarized PVDF 5050P fibers showed higher static charges with lower fluctuations due to the enhanced β-phase and uniform dipole distribution; whereas the CH_2_-CF_2_ dipoles were randomly oriented over the plane of the fiber mat by electrically aided mechanical stretching of the jet during electrospinning. In our earlier work on non-polarized PVDF fibers, we reported real-time atomic-resolution electron micrographs that clearly revealed the paths of unoriented CF_2_ dipoles perpendicular to the view direction [21].

The porosities of the fiber mats in Table 2 were measured by a custom-made pycnometer, as described in [6]. The polarization of the fiber mats caused some of the fibers to be attracted to each other and enlarged the pores in the mat. This resulted in larger pores and, hence, higher permeability (lower pressure drop) for the gas to flow through the fiber mats.

### 3.2. TSI8130: Filter Media Test Results

A custom-made Plexiglas filter holder was used to hold the fiber mats with their surfaces perpendicular to the flow direction of the aerosol mixture. Fiber mats supported by a stainless steel mesh with openings of approximately 5 mm in diameter (see Figure S1). The supporting mesh was tested without a fiber mat to ensure it had no effect on particle capturing performance and negligible pressure drop.

**Figure 7:**
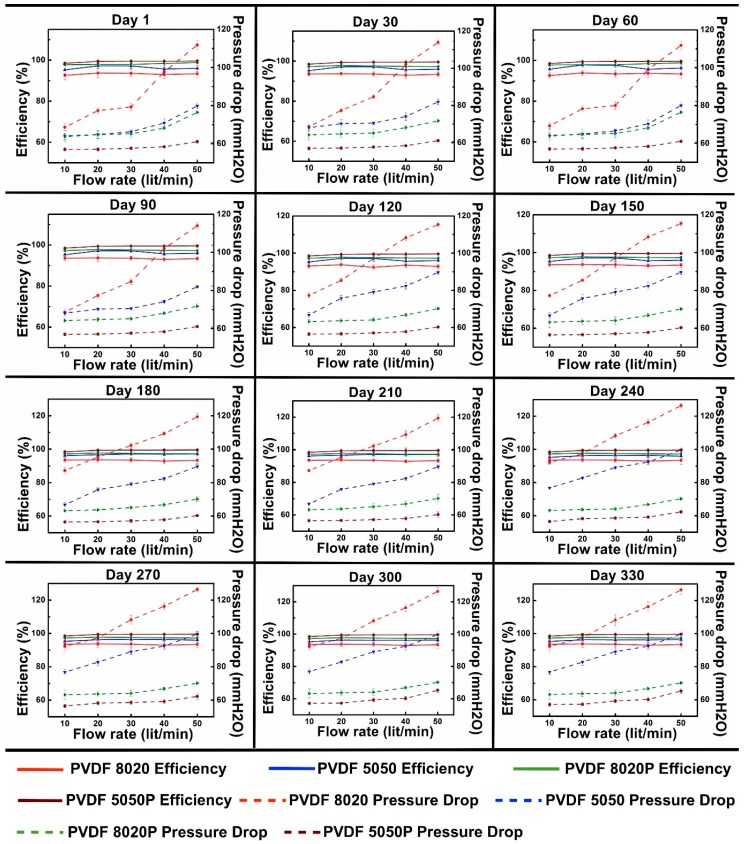
Performance profiles of particle capture efficiency (%E, solid lines) and pressure drop (ΔP, dotted lines) of PVDF fiber before and after polarization with time.

Figure 7 shows the filtration performance with particle loading on four different filter media samples with five different flowrates for twelve experiments conducted on the same fiber samples conducted sequentially in 30-day increments. Each plot shows the particle capture efficiency and pressure drop across the fiber samples at different flow rates using the TSI8130. Each subsequent test was conducted with the particles captured in the prior tests still in the fiber mat samples. As a result, the mats tested on Day 330 were cumulatively challenged with twelve times as much total gas flow and total particles as compared to the mats tested on Day 1.

The data in Figure 7 show that the polarized fiber mats had higher capture efficiencies and lower pressure drops than the unpolarized fiber mats, as expected, due to the electrostatic capture mechanism and the larger pores of the polarized mats. The 5050 and 5050P mats had higher efficiencies than the 8020 and 8020P mats, respectively, due to the smaller fibers and higher surface areas of the 5050 mats.

The polarized mats had lower pressure drops than the unpolarized mats due to the larger pores, even with particle loading. The pressure drops for all of the mats increased roughly linearly as the flow rate increased. In general, the pressure drops tended to increase with the test day due to the loading of the fiber mats with captured particles over time. The change in pressure drop was most notable for the unpolarized mats, suggesting that the polarized and unpolarized mats may retain the captured particles differently.

**Figure 8:**
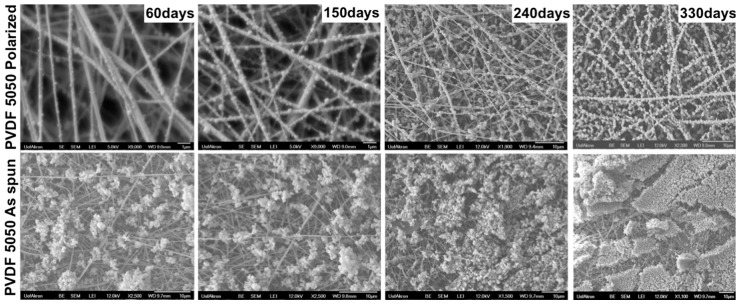
SEM images showing NaCl particles captured on PVDF 5050 polarized and PVDF 5050 as-spun filter media after 60, 150, 240 and 330 days. Images reported in the top row are PVDF 5050P polarized media, and the bottom row are PVDF 5050 as-spun media.

SEM images were taken of the surfaces of the mats after each experiment. Figure 8 shows images taken at 60, 150, 240 and 330 days. The images clearly show that the particles in the polarized fiber mats tend to stick directly to the fibers, leaving most of the pore spaces open for flow. This is in contrast to the non-polarized fiber mats, where many, if not most, of the particles tended to be captured onto other prior captured particles, thus forming dendrites, clusters and eventually cakes of particles captured in the pores and on the mat surface. The mats were very thin, on the order of tenth of a mm; hence, the variation of particle capture through the depth of the mats was difficult to observe.

**Figure 9:**
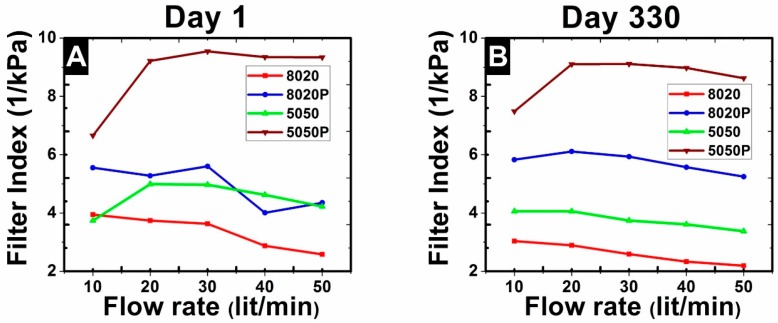
Filtration index (FI) value comparisons of different filters at Day 1 (**A**) and Day 330 (**B**).

Filter performance may be characterized by the filtration index [49] or quality factor [50], calculated by the expression, where $C_{in}$ and $C_{out}$ are the inlet and outlet particle concentrations as the flow passed through the fiber mat and ΔP is the pressure drop. The filtration index characterizes the filter separation performance relative to the effort (pressure drop) required to achieve the separation. The higher the filtration index, the better performance of the filter. The plots of filtration index in Figure 9 for Days 1 and 330 show that the 5050P mats performed the best at all flow rates. Initially, the 8020P and the 5050 mats performed about the same on Day 1, but on Day 330, the 8020P mats performed better than the 5050 mats. The filtration indexes tended to decrease moderately as the flow rate increased.

(1) $$Filtration\;Index=\;\frac{-\text{ln}\left(\frac{C_{out}}{C_{in}}\right)}{\Delta P}$$

## 4. Summary and Conclusions

In this work, bead-free PVDF fibers were electrospun under a controlled process and solution conditions. PVDF 5050 fibers with diameters less than 200 nm showed high crystallinity with higher content of the β-phase than non-polarized fibers. The fiber mats were polarized by heating and stretching in an electric field. The polarized mats had larger pores than the non-polarized 5050 mats. The 5050 polarized mats had higher electrostatic charges due to the internal orientations of the β-phase crystalline structures. The PVDF 5050 polarized fiber mats had higher capture efficiencies, lower pressure drops and higher filtration indexes than non-polarized mats over time with repeated experiments and particle loading. The electrostatic mechanism of the particle capture of the 5050 polarized mats dominated the particle capture as indicated by SEM images, which showed captured particles distributed as many individual particles attached to many fibers. In comparison, the particles captured by non-polarized 5050 fiber mats tended to form internal and surface cakes of particles. The larger pores of the 5050 polarized mats and the distribution of the captured particles over the surfaces of the fibers contributed to the lower pressure drops. The strong electrostatic forces contributed to the high capture efficiencies of these media.

This work shows that electrospun polarized PVDF fiber mats can function effectively as an air filter. This work should be expanded in the future to investigate their performance with other types of particles and aerosols. These materials should be investigated for their performance in applications, such as face masks, cabin air filters and in industrial applications. Experiments are needed to determine the loading capacity and the shelf life of the electrostatic charge effects.

## Figures and Tables

**Table 1:** Electrospinning conditions of PVDF fibers.

Polymer Solution Concentration (wt./wt. %)	Solvent Ratio (Acetone:DMF)	TFA Concentration in Solution (wt./wt. %)	Applied Voltage (kV)	Tip to Collector Distance (cm)	Drum Rotation Speed (RPM)	Syringe Flow Rate (mL/h)	Fiber Diameter Range (nm)	Average Fiber Diameter (nm)	Fiber Diameter Standard Deviation (nm)
20%	80:20	0	20	12	100	15	1800–2900	2037	92
10%	50:50	3%	17	20	100	5	5–293	197	87

**Table 2:** An empirical comparison of porosity, surface textural and thermal data of electrospun PVDF fibers before and after polarization.

Type of Fiber Sample	Porosity (ε) by Gas Expansion Method	Porosity (ε) by Mass Balance Method	DSC-Crystallinity X_C_ (%)	DSC-Crystallization Temperature T_C,P_ (°C)	TGA-CH_2_ Degradation Peak (°C)	TGA-CF_2_ Degradation Peak (°C)	BET Surface Area (M^2^/g)	Average Surface Pore Size (Nm)
PVDF 8020	0.89 ± 0.24	0.92 ± 0.22	49.3 ± 2.32	128.9 ± 2.32	407.1 ± 2.31	426.1 ± 2.47	36.48 ± 1.23	13.16 ± 1.26
PVDF 5050	0.92 ± 0.75	0.94 ± 0.23	57.7 ± 1.73	127.2 ± 1.49	412.3 ± 3.12	428.2 ± 2.12	42.57 ± 2.07	6.72 ± 1.33
PVDF 8020P	0.90 ± 0.66	0.98 ± 0.53	69.6 ± 1.89	123.7 ± 3.36	411.6 ± 1.25	492.0 ± 2.19	35.21 ± 2.16	19.47 ± 2.09
PVDF 5050P	0.94 ± 0.02	0.99 ± 0.42	79.2 ± 1.79	123.4 ± 2.22	416.7 ± 3.14	503.4 ± 2.37	41.26 ± 1.21	11.21 ± 1.92

## Supplementary Materials

The following are available online at www.mdpi.com/1996-1944/9/8/671/s1.

## References

[1] Bernstein J.A., Alexis N., Barnes C., Bernstein I.L., Nel A., Peden D., Diaz-Sanchez D., Tarlo S.M., Williams P.B., Bernstein J.A. (2004). Health effects of air pollution. J. Allergy Clin. Immunol..

[2] Kampa M., Castanas E. (2008). Human health effects of air pollution. Environ. Pollut..

[3] Pope C.A., Dockery D.W. (2006). Health effects of fine particulate air pollution: Lines that connect. J. Air Waste Manag. Assoc..

[4] Pope C.A., Bates D.V., Raizenne M.E. (1995). Health effects of particulate air pollution: Time for reassessment?. Environ. Health Perspect..

[5] Seaton A., MacNee W., Donaldson K., Godden D. (1995). Particulate air pollution and acute health effects. Lancet.

[6] Yang X., Wang H., Chase G.G. (2015). Performance of Hydrophilic Glass Fiber Media to Separate Dispersed Water Drops from Ultra Low Sulfur Diesel Supplemented by Vibrations. Sep. Purif. Technol..

[7] Patel S.U., Manzo G.M., Patel S.U., Kulkarni P.S., Chase G.G. (2012). Permeability of electrospun superhydrophobic nanofiber mats. J. Nanotechnol..

[8] Viswanadam G., Chase G.G. (2013). Water-diesel secondary dispersion separation using superhydrophobic tubes of nanofibers. Sep. Purif. Technol..

[9] Patel S.U., Chase G.G. (2014). Separation of water droplets from water-in-diesel dispersion using superhydrophobic polypropylene fibrous membranes. Sep. Purif. Technol..

[10] Davoudi M., Fang J., Chase G.G. (2016). Barrel shaped droplet movement at junctions of perpendicular fibers with different orientations to the air flow direction. Sep. Purif. Technol..

[11] Doshi J., Reneker D.H. (1995). Electrospinning process and applications of electrospun fibers. J. Electrost..

[12] Shin H.U., Lolla D., Nikolov Z., Chase G.G. (2015). Pd-Au Nanoparticles Supported by TiO_2_ Fibers for Catalytic NO Decomposition by CO. J. Ind. Eng. Chem..

[13] Rajala J., Shin H., Lolla D., Chase G. (2015). Core-Shell Electrospun Hollow Aluminum Oxide Ceramic Fibers. Fibers.

[14] Reneker D.H., Chun I. (1999). Nanometre diameter fibres of polymer, produced by electrospinning. Nanotechnology.

[15] Lin Y., Clark D.M., Yu X., Zhong Z., Liu K., Reneker D.H. (2012). Mechanical properties of polymer nanofibers revealed by interaction with streams of air. Polymer.

[16] Thompson C.J., Chase G.G., Yarin A.L., Reneker D.H. (2007). Effects of parameters on nanofiber diameter determined from electrospinning model. Polymer.

[17] Shin H.U., Li Y., Paynter A., Nartetamrongsutt K., Chase G.G. (2015). Vertical rod method for electrospinning polymer fibers. Polymer.

[18] Miao J., Bhatta R.S., Reneker D.H., Tsige M., Taylor P.L. (2014). Molecular dynamics simulations of relaxation in stretched PVDF nanofibers. Polymer.

[19] Wisniewski C., Ferreira G.F.L., Moura W.A., Giacometti J.A., Wisniewski C., Ferreira G.F.L. (2000). Study of ferroelectric polarization in poly(vinylidene fluoride) using the constant current method. J. Phys. D Appl. Phys..

[20] Ma X., Liu J., Ni C., Martin D.C., Chase D.B., Rabolt J.F. (2012). Molecular orientation in electrospun poly(vinylidene fluoride) fibers. ACS Macro Lett..

[21] Lolla D., Gorse J., Kisielowski C., Miao J., Taylor P.L., Chase G.G., Reneker D.H. (2016). Polyvinylidene fluoride molecules in nanofibers, imaged at atomic scale by aberration corrected electron microscopy. Nanoscale.

[22] Lovinger A.J. (1983). Ferroelectric Polymers. Science.

[23] Baji A., Mai Y.-W., Li Q., Liu Y. (2011). Electrospinning induced ferroelectricity in poly(vinylidene fluoride) fibers. Nanoscale.

[24] Bormashenko Y., Pogreb R., Stanevsky O., Bormashenko E. (2004). Vibrational spectrum of PVDF and its interpretation. Polym. Test..

[25] Ahmed B., Raghuvanshi S.K., Sharma N.P., Krishna J.B.M., Wahab M.A. (2013). 1.25 MeV Gamma Irradiated Induced Physical and Chemical Changes in Poly Vinylidene Fluoride (PVDF) Polymer. Prog. Nanotechnol. Nanometer.

[26] Lanceros-Méndez S., Mano J.F., Costa A.M., Schmidt V.H. (2001). Ftir and DSC Studies of Mechanically Deformed Β-Pvdf Films. J. Macromol. Sci. Part B.

[27] Ignatova M., Yovcheva T., Viraneva A., Mekishev G., Manolova N., Rashkov I. (2008). Study of charge storage in the nanofibrous poly(ethylene terephthalate) electrets prepared by electrospinning or by corona discharge method. Eur. Polym. J..

[28] Ali Kilic B.-Y.Y., Shim E., Pourdeyhimi B. (2014). Aerosol Filtration Properties of Nucleating Agent Containing Electret Filters. Polym. Eng. Sci..

[29] Walls H.J., Ensor D.S., Andrady A.L., Walker T.A. (2009). Particle Filter System Incorporating Electret Nanofibers. U.S. Patent No..

[30] Cho D., Naydich A., Frey M.W., Joo Y.L. (2013). Further improvement of air filtration efficiency of cellulose filters coated with nanofibers via inclusion of electrostatically active nanoparticles. Polymer.

[31] Wang C.-S. (2001). Electrostatic forces in fibrous filters—A review. Powder Technol..

[32] Dargaville T.R.T., Celina M.C., Elliot J., Chaplya P.M., Elliott J.M., Jones G.D., Mowery D.M., Assink R.A., Clough R.L., Martin J.W. (2005). Characterization, Performance and Optimization of PVDF as a Piezoelectric Film for Advanced Space Mirror Concepts.

[33] Tsai P.P., Schreuder-Gibson H., Gibson P. (2002). Different electrostatic methods for making electret filters. J. Electrostat..

[34] Goel M. (2003). Electret sensors, filters and MEMS devices: New challenges in materials research. Curr. Sci..

[35] Iverson W.P. (1985). Separator for separating fluid media from minute particles of impurities. U.S. Patent No..

[36] Emi H., Wang C. (1982). Filtration Model Filters. AlChE J..

[37] Kilic A., Shim E., Yeom B.Y., Pourdeyhimi B. (2013). Improving electret properties of PP filaments with barium titanate. J. Electrostat..

[38] Hutten I.M. (2016). Handbook of Nonwoven Filter Media.

[39] Salimi A., Yousefi A.A. (2003). Analysis Method. Polym. Test..

[40] Khayet M., Khulbe K., Matsuura T. (2004). Characterization of membranes for membrane distillation by atomic force microscopy and estimation of their water vapor transfer coefficients in vacuum membrane distillation process. J. Memb. Sci..

[41] Lee H., Cooper R., Wang K., Liang H. (2008). Nano-Scale Characterization of a Piezoelectric Polymer (Polyvinylidene Difluoride, PVDF). Sensors.

[42] Barhate R.S., Ramakrishna S. (2007). Nanofibrous filtering media: Filtration problems and solutions from tiny materials. J. Memb. Sci..

[43] Manzo G.M., Wu Y., Chase G.G., Goux A. (2016). Comparison of nonwoven glass and stainless steel microfiber media in aerosol coalescence filtration. Sep. Purif. Technol..

[44] Nartetamrongsutt K., Chase G.G. (2013). The influence of salt and solvent concentrations on electrospun polyvinylpyrrolidone fiber diameters and bead formation. Polymer.

[45] Zheng J., Zhuang M., Yu Z., Zheng G., Zhao Y., Wang H., Sun D. (2014). The Effect of Surfactants on the Diameter and Morphology of Electrospun Ultrafine Nanofiber. J. Nanomater..

[46] Li Y., Zhang X., Trudick E., Chase G.G. (2015). Simulation of electrostatic field in electrospinning of polymer nanofibers. Nanoscale Syst. Math. Model. Theory Appl..

[47] Rengasamy S., Eimer B.C., Shaffer R.E. (2009). Comparison of Nanoparticle Filtration Performance of NIOSH-approved and CE-Marked Particulate Filtering Facepiece Respirators. Ann. Occup. Hyg..

[48] Shaffer R.E., Rengasamy S. (2009). Respiratory protection against airborne nanoparticles: A review. J. Nanopart. Res..

[49] Westfall C. (2012). How Argonne’s Intense Pulsed Neutron Source Came to Life and Gained Its Niche: The View from an Ecosystem Perspective. Argonne Natl. Lab..

[50] Brown R.C. (1993). Air Filtration: An Integrated Approach to the Theory and Application of Fibrous Filters.

