# Supplementary material for: Fabrication, Polarization of Electrospun Polyvinylidene Fluoride Electret Fibers and Effect on Capturing Nanoscale Solid Aerosols
Source: Materials (Basel). 2016 Aug 9;9(8):671. doi: 10.3390/ma9080671 (PMC5510728; doi:10.3390/ma9080671)
Supplement: Supplementary file 1 [file materials-09-00671-s001.pdf]

Supplementary Materials: Fabrication, Polarization of Electrospun Polyvinylidene Fluoride Electret Fibers and Effect on Capturing Nanoscale Solid Aerosols

Dinesh Lolla, Manideep Lolla, Ahmed Abutaleb, Hyeon U. Shin, Darrell H. Reneker and George G. Chase

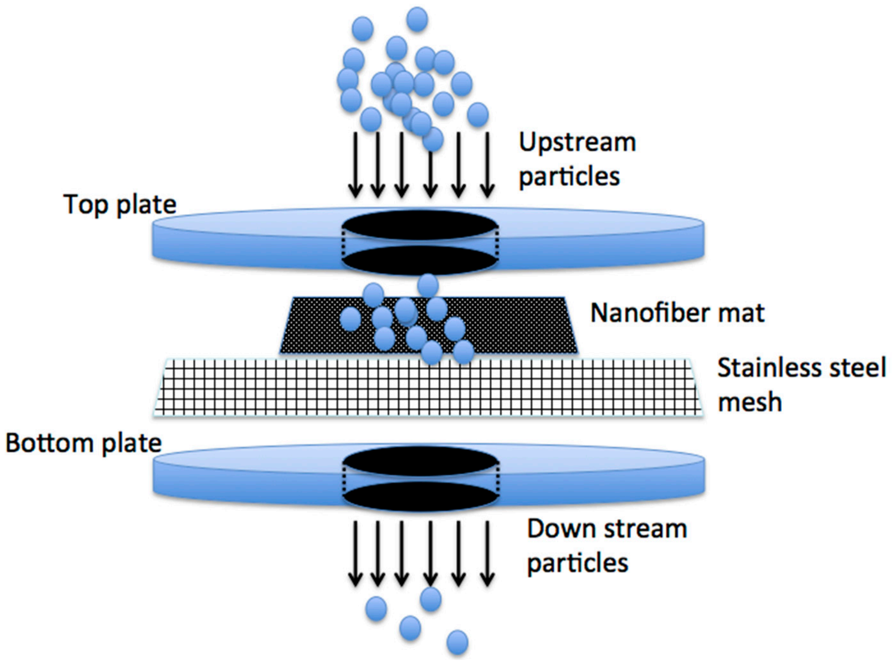

Figure S1. Schematic for the filter holder used for performing the TSI 8130 experiments. The nanofiber mats and stainless steel mesh were sandwiched between two Plexiglas discs with 5.7 cm diameter holes in the center.

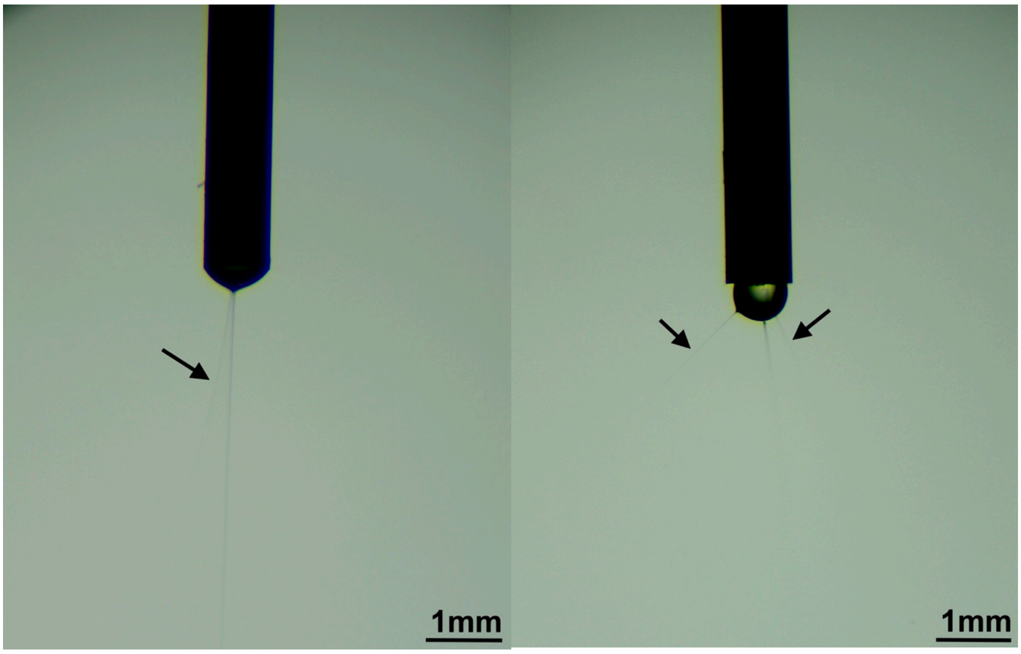

Figure S2. High magnification microscopic image of the electrospinning needle showing the formation of very fine jets. These finer jets were expected to be the potential reason for the formation of fibers with diameters of less than 50 nm. Arrows indicate secondary jets.

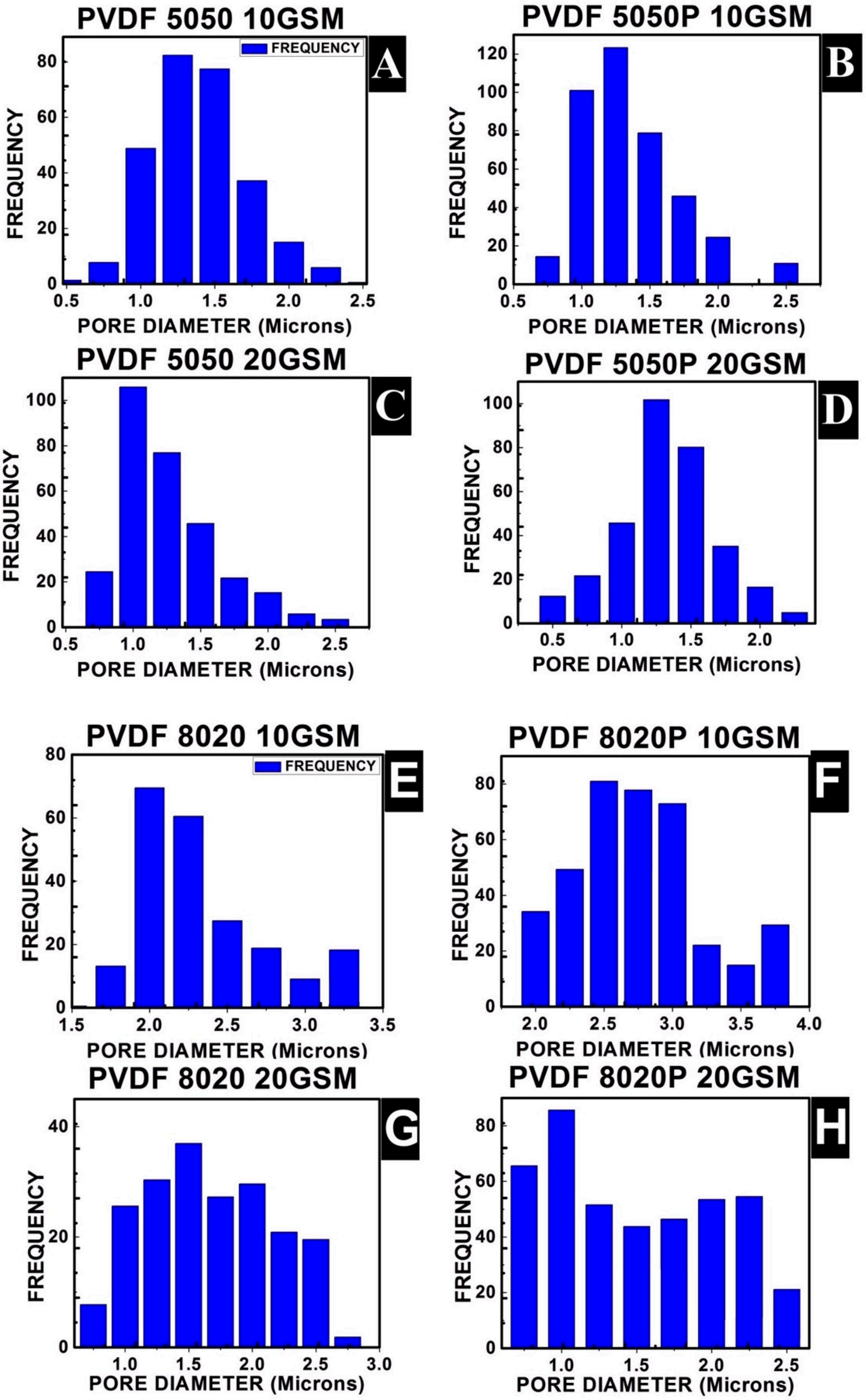

Figure S3. Pore size distributions of filter samples with different basis weights (GSM = grams/m^2) (**A**) PVDF 5050 10GSM; (**B**) PVDF 5050P 10GSM; (**C**) PVDF 5050 20GSM; (**D**) PVDF 5050P 20GSM; (**E**) PVDF 8020 10GSM; (**F**) PVDF 8020P 10GSM; (**G**) PVDF 8020 20GSM and (**H**) PVDF 8020P 20GSM. Note: fibers with a 10GSM basis weight were not used in the filtration experiments.

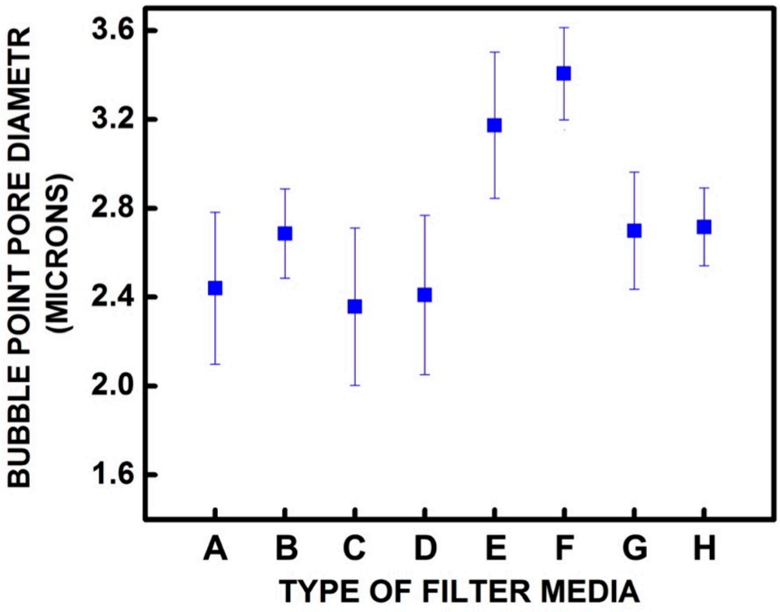

Figure S4. Bubble point diameters (i.e., largest flow-through pore diameter) for mats of different average fiber diameters and basis weights corresponding to the size distributions in Figure 3. The data points are the averages of at least three samples, and the error bars represent one standard deviation.

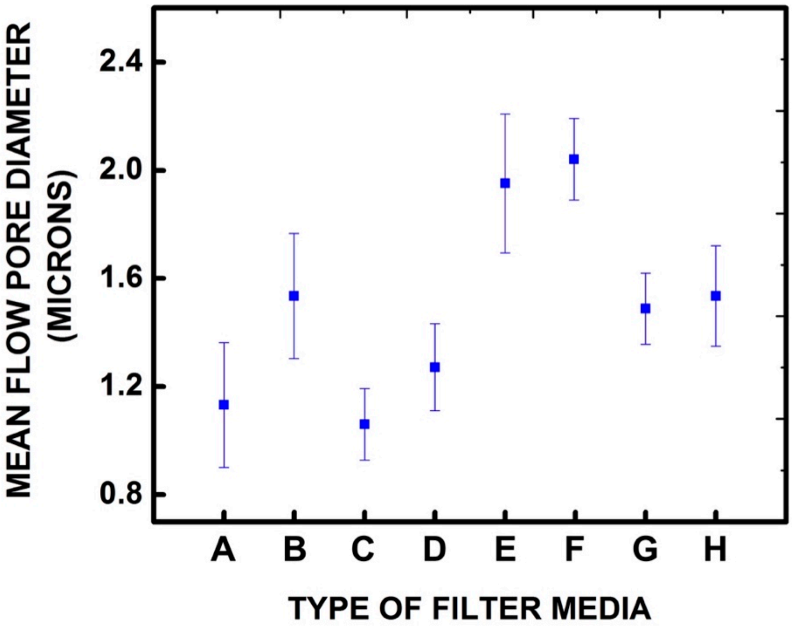

Figure S5. Mean flow pore diameters for different average fiber diameters and basis weights corresponding to the size distributions in Figure 3. The data points are the averages of at least three samples, and the error bars represent one standard deviation.
